# Ergothioneine attenuates whole-abdominal irradiation-induced multi-organ injury via the gut-heart-brain axis by modulating calcium voltage-gated channel subunit alpha1 C (Cacna1c) expression

**DOI:** 10.1186/s43556-025-00402-3

**Published:** 2026-01-14

**Authors:** Xudong Ding, Jia Du, Zhaoyu Wang, Lu Lu, Saijun Fan

**Affiliations:** Institute of Radiation Medicine, Chinese Academy of Medical Sciences and Peking Union Medical College, Tianjin Key Laboratory of Radiation Medicine and Molecular Nuclear Medicine, State Key Laboratory of Advanced Medical Materials and Devices, Tianjin, 300192 China

**Keywords:** Ionizing radiation, Ergothioneine, Intestinal, Heart, Cognitive function

## Abstract

Despite the precise targeting of radiation therapy, collateral damage to adjacent healthy tissues remains an inevitable consequence. Currently, no effective clinical intervention exists to prevent or alleviate these adverse effects. To address this issue, our study established a whole-abdominal irradiation (WAI) model using C57BL/6J mice to investigate the systemic effects of ionizing radiation (IR) on the gut and other organs. Results confirmed that IR not only causes significant intestinal damage but also induces cardiac injury and cognitive dysfunction through remote effects. Ergothioneine (EGT), a naturally occurring dietary sulfur compound, has garnered significant attention in recent years for its unique functions in antioxidant, anti-inflammatory, and metabolic regulation. Our findings reveal that EGT significantly mitigates IR-induced structural damage to the intestine, preserves crypt-villus architecture, restores goblet cell numbers, and reduces systemic inflammation. Furthermore, EGT modified post-IR gut microbiota composition by decreasing the relative abundance of *Candidatus_Soleaferrea* and downregulating calcium voltage-gated channel subunit alpha1 C (Cacna1c) expression. EGT alleviated WAI-induced cardiac and cognitive dysfunction through the gut-heart and gut-brain axes. EGT also ameliorated dextran sulfate sodium (DSS)-induced colitis and enhanced intestinal barrier function. Our findings identify EGT as a novel multi-organ radioprotective agent that acts through microbiota modulation and Cacna1c regulation, providing a viable strategy for improving radiotherapy safety and efficacy.

**Supplementary Information:**

The online version contains supplementary material available at 10.1186/s43556-025-00402-3.

## Introduction

Radiation therapy is a fundamental treatment for many types of cancer, used in over 50% of cases [1]. However, IR therapy inevitably damages peritumoral healthy tissues, thereby inducing adverse effects that ultimately compromise therapeutic outcomes and patient quality of life [2, 3]. During abdominal or pelvic radiotherapy, the high radiosensitivity of the intestines often results in barrier dysfunction and microbial dysbiosis, thereby precipitating IR enteritis. This condition manifests clinically as nausea, diarrhea, and infection, with severe cases potentially becoming life-threatening [4, 5]. Furthermore, abdominal radiotherapy may elicit off-target cardiac complications through abscopal effects, including cardiomyopathy and conduction abnormalities [6, 7], as well as neurocognitive impairment via mechanisms like the miR-34a-5p/BDNF axis [8, 9]. Consequently, there is an urgent clinical need to develop effective strategies that mitigate these detrimental sequelae.

EGT is a naturally occurring dietary thiol compound primarily biosynthesized by fungi and actinomycetes. It demonstrates favorable bioavailability and a pronounced capacity for tissue accumulation in humans, underscoring its potential in health maintenance and metabolic regulation [10]. Owing to its potent antioxidant and anti-inflammatory properties, EGT has demonstrated efficacy in ameliorating conditions such as DSS-induced colitis and anthracycline-related cardiotoxicity [11, 12]. Furthermore, its neuroprotective efficacy extends to mitigating cognitive deficits and conferring antiepileptic effects [13]. Nevertheless, the mechanisms underlying EGT's protective role against IR-induced multi-organ injury remain incompletely understood.

Emerging evidence underscores the pivotal roles of the gut-heart and gut-brain axes in systemic physiological regulation. Gut microbiota-derived metabolites, such as trimethylamine N-oxide (TMAO) and short-chain fatty acids (SCFAs), contribute to the pathogenesis of cardiovascular diseases by modulating inflammatory responses, lipid metabolism, and endothelial function [14, 15]. Similarly, gut microbes participate in central nervous system regulation via the gut-brain axis [16]. IR-induced intestinal injury is primarily driven by oxidative stress and DNA damage, triggering disruption of the epithelial barrier, which in turn leads to microbial dysbiosis [17]. However, it remains unclear to what extent such intestinal injuries subsequently mediate cardiac and cognitive dysfunction via these pathways.

Based on existing evidence, we hypothesized that EGT could mitigate multi-organ injury after abdominal irradiation by modulating gut microbiota and key signaling pathways through the gut-heart and gut-brain axes. To test this hypothesis, we systematically evaluated the protective effects of EGT against WAI-induced intestinal, cardiac, and cognitive dysfunction. Our results demonstrate that EGT not only alleviates intestinal barrier disruption and systemic inflammation but also reduces the abundance of the *Candidatus_Soleaferrea*, while concurrently ameliorating myocardial injury and cognitive deficits. Importantly, this study is the first to elucidate the mechanism by which EGT functions as a multi-organ radioprotector via regulation of Cacna1c and gut-organ axes thereby offering a promising translational strategy for the clinical prevention and treatment of radiotherapy-related tissue damage.

## Results

### EGT ameliorates WAI-induced intestinal injury and inflammation in male mice

**Fig. 1:**
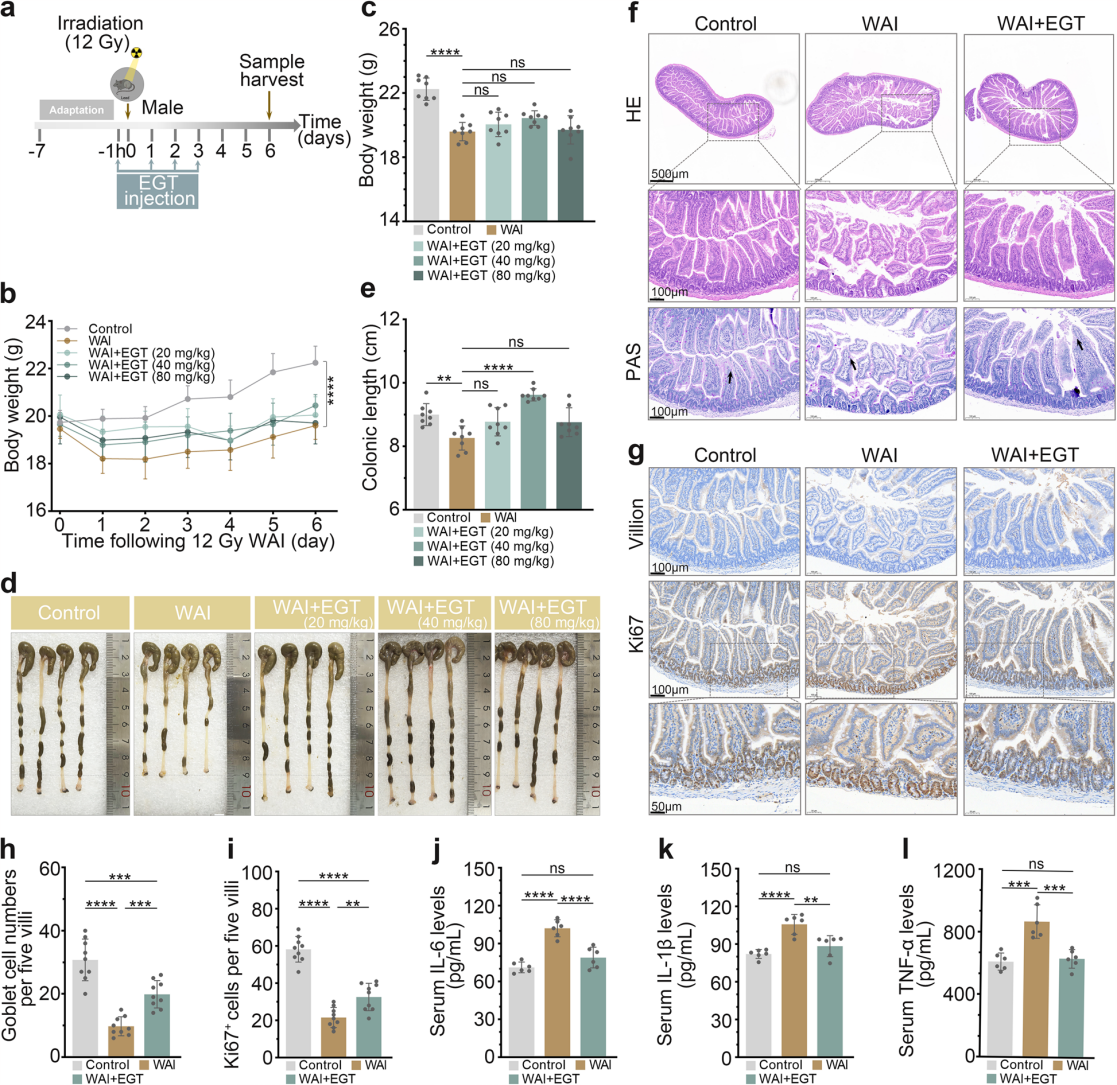
EGT protects against WAI-induced intestinal injury. **a** Experimental timeline of 12 Gy WAI and EGT treatment. **b**-**c** EGT attenuated WAI-induced body weight loss (*n* = 8). Representative colon images (**d**) and length quantification (**e**) (*n* = 8). **f** Representative images of small intestine tissue H&E and PAS staining (scale bar, 500 µm and 100 µm). **g** IHC detection of Ki67 and Villin in intestinal tissue (scale bar, 100 µm and 50 µm). Quantification of goblet cells (**h**) and Ki67^+^ cells (**i**) per five villi (*n* = 9). Serum concentrations of IL-6 (**j**), IL-1β (**k**), and TNF-α (**l**) (*n* = 6). Data are presented as mean ± SD; ^**^P < 0.01, ^***^P < 0.001, ^****^P < 0.0001

To assess the radioprotective effect of EGT on WAI-induced intestinal injury in male mice, the animals received 12 Gy WAI and were subsequently treated with intraperitoneal injections of EGT at doses of 20, 40, and 80 mg/kg (Fig. 1a). While WAI caused significant body weight loss that was not mitigated by EGT treatment (Fig. 1b, c). We observed a dose-dependent protective effect of EGT against IR-induced colon shortening, with the 40 mg/kg dose exhibiting the most pronounced efficacy in restoring normal colon length (Fig. 1d, e). H&E and PAS staining revealed severe intestinal injury in WAI mice, characterized by villus destruction, crypt loss, and decreased goblet cells (Fig. 1f, h). EGT treatment markedly preserved intestinal architecture, maintaining crypt-villus integrity and restoring goblet cell numbers. Furthermore, immunohistochemistry (IHC) analysis demonstrated that WAI significantly reduced the abundance of both Ki67^+^ proliferating cells and Villin^+^ differentiated epithelial cells. Importantly, EGT administration effectively restored these cellular populations, suggesting an enhanced regenerative capacity of the intestinal epithelium following IR (Fig. 1g, i) [18]. Based on serum analysis of key inflammatory mediators IL-6、IL-1β and TNF-α, EGT significantly attenuated systemic inflammation induced by WAI (Fig. 1j-l). Collectively, these findings demonstrate that EGT effectively ameliorates both intestinal structural injury and systemic inflammatory responses in male mice following WAI exposure.

### EGT reshapes WAI-induced gut microbiota dysbiosis

**Fig. 2:**
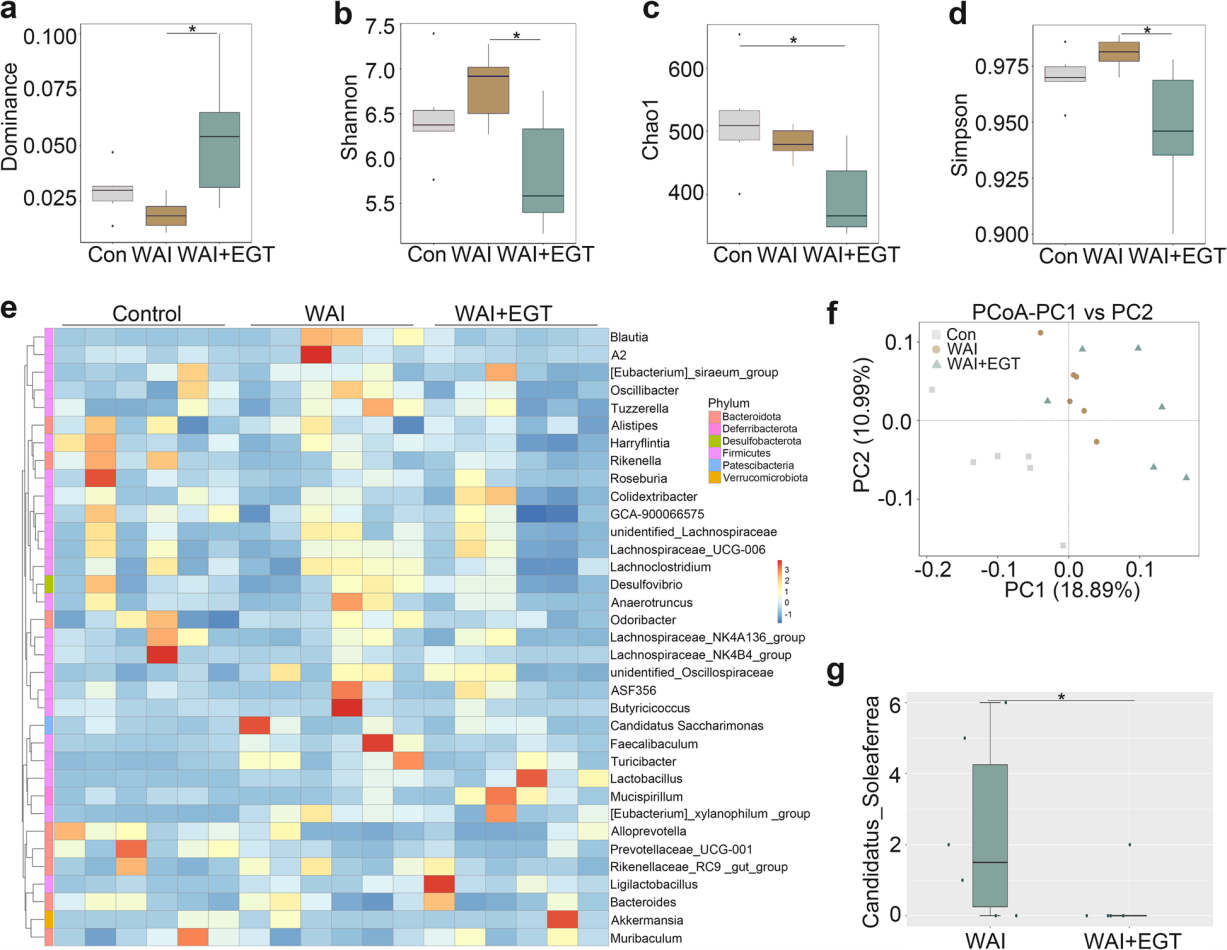
EGT modulates gut microbiota composition post-WAI. Alpha diversity analysis showing changes in Dominance (**a**), Shannon (**b**), Chao1 (**c**), and Simpson (**d**) indices in male mice. **e** Genus-level clustering heatmap of microbial communities. **f** Beta diversity PCoA plot demonstrating microbiota structural differences. **g** Relative abundance of *Candidatus_Soleaferrea*. Data are shown as the means ± SD;.^*^P < 0.05

Emerging evidence suggests that IR-induced microbial dysbiosis plays a pivotal role in the progression of intestinal injury [19]. To investigate whether EGT exerts its protective effect through modulation of the gut microbiota, we performed 16S rRNA gene sequencing on fecal samples from different treatment groups. Analysis of alpha-diversity demonstrated that EGT treatment significantly enhanced microbial community evenness, as reflected by the Dominance, Simpson and Shannon indices, but did not significantly alter species richness according to the Chao1 index (Fig. 2a-d), This indicates that EGT treatment primarily modulates gut microbial community structure by enhancing species evenness and significantly reducing the abundance of *Candidatus_Soleaferrea*, rather than increasing overall species richness. Beta diversity analysis revealed distinct clustering patterns, with principal coordinate analysis (PCoA) showing significant separation between groups (Fig. 2f), notably, hierarchical clustering demonstrated that EGT treatment restored the relative abundance of multiple bacterial genera to near-control levels (Fig. 2e). Of particular interest, EGT significantly attenuated the IR-induced proliferation of *Candidatus_Soleaferrea* (Fig. 2g), which is a pro-inflammatory pathobiont previously linked to intestinal barrier dysfunction [20]. In summary, EGT treatment substantially reshaped the gut microbial composition after WAI exposure, with a marked reduction in the abundance of the *Candidatus_Soleaferrea*.

### EGT ameliorates WAI-induced intestinal injury through targeted downregulation of Cacna1c

**Fig. 3:**
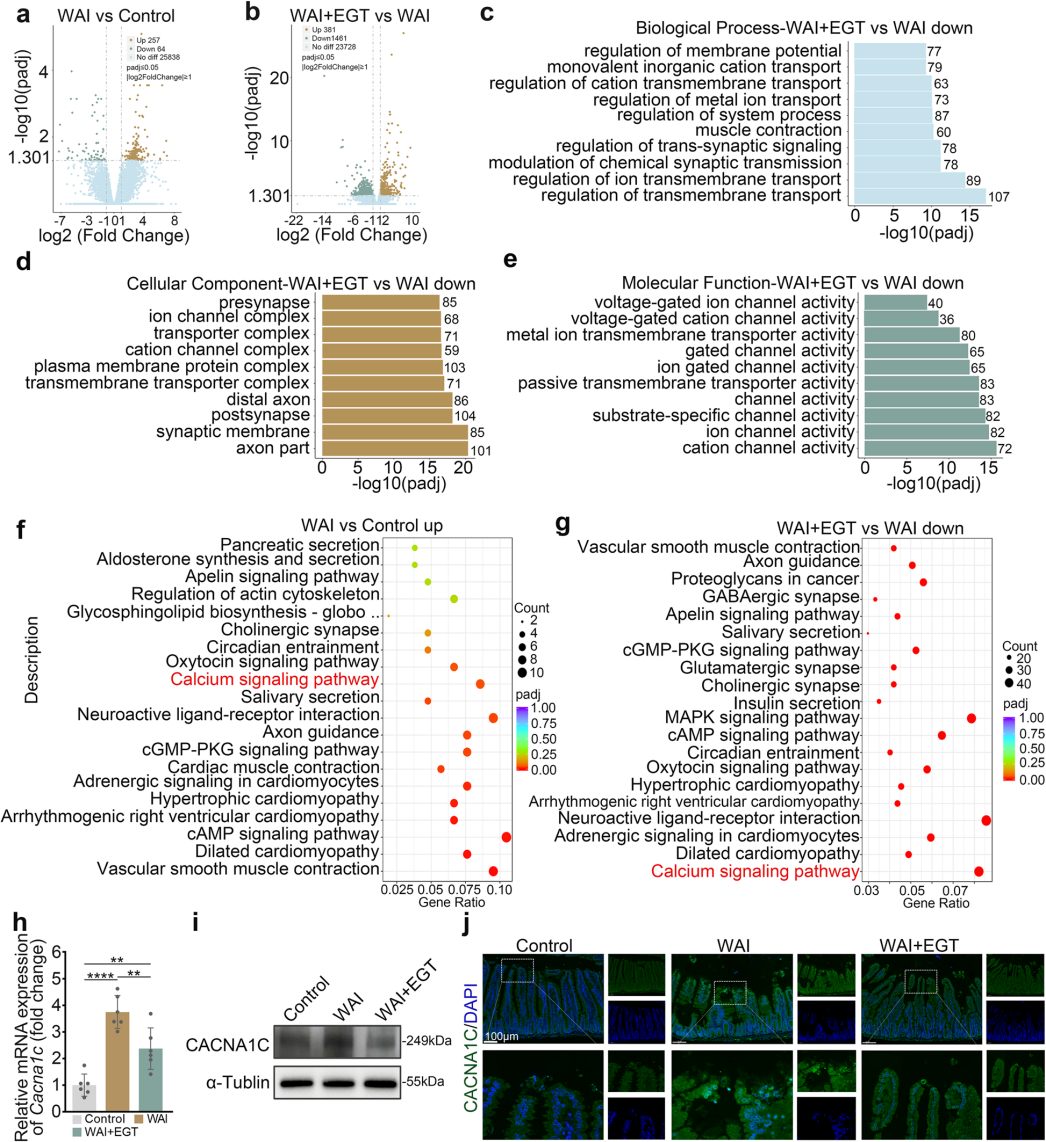
EGT exerts radiation protection through Cacna1c. **a** Differential gene expression in small intestinal tissue between the control group and the WAI group. **b** Differential gene expression in small intestinal tissue between the WAI group and the WAI + EGT group. GO enrichment analysis of Biological Processes (**c**), Cellular Components (**d**), and Molecular Functions (**e**) in WAI + EGT vs WAI. **f** KEGG pathway enrichment analysis between the control group and the WAI group. **g** KEGG pathway enrichment analysis between the WAI group and the WAI + EGT group. **h** qRT-PCR validation of mRNA expression (*n* = 6). **i** Protein level of CACNA1C. **j** Representative IHC images of Cacna1c expression (scale bar, 100 µm). Data are shown as the means ± SD; ^**^P < 0.01, ^***^P < 0.0001

To elucidate the molecular mechanisms underlying EGT's radioprotective effects, we profiled the transcriptomes of mouse small intestinal tissues using RNA-seq. Compared to controls, WAI induced 257 upregulated and 64 downregulated genes, whereas EGT treatment elicited more extensive transcriptional changes with 381 upregulated and 1,461 downregulated genes relative to WAI (Fig. 3a, b). Gene Ontology (GO) enrichment analysis demonstrated that radiation-altered genes were predominantly associated with muscle system processes and calcium channel activity, particularly localized to calcium channel complexes (Fig. S1a-c). Strikingly, EGT treatment specifically restored expression patterns of genes involved in calcium ion transport and channel function (Fig. 3c-e). These findings collectively indicate that EGT's radioprotective effects are mediated through modulation of calcium channel-related signaling pathways in intestinal tissue. Kyoto Encyclopedia of Genes and Genomes (KEGG) pathway enrichment analysis revealed distinct biological patterns between IR-induced and EGT-modulated gene expression. IR-triggered gene upregulation was primarily linked to vascular smooth muscle contraction and dilated cardiomyopathy pathways (Fig. 3f), whereas EGT-mediated downregulation preferentially affected calcium signaling and cardiomyopathy-related pathways (Fig. 3g). Based on KEGG and GO analysis results, we further screened key differentially expressed genes and found that *Cacna1c* exhibited significant expression changes. Subsequent validation of *Cacna1c* expression by qRT-PCR and of CACNA1C levels by Western blot and IHC confirmed the RNA sequencing results (Fig. 3h-j). Consistently across these methods, EGT treatment effectively downregulated the elevated expression of Cacna1c induced by WAI. These findings confirm that the specific regulation of Cacna1c is a key mechanism by which EGT exerts its radiation protection effects.

### EGT attenuates WAI-induced cardiomyopathy through gut-heart axis modulation by targeting Cacna1c-mediated calcium signaling pathways

**Fig. 4:**
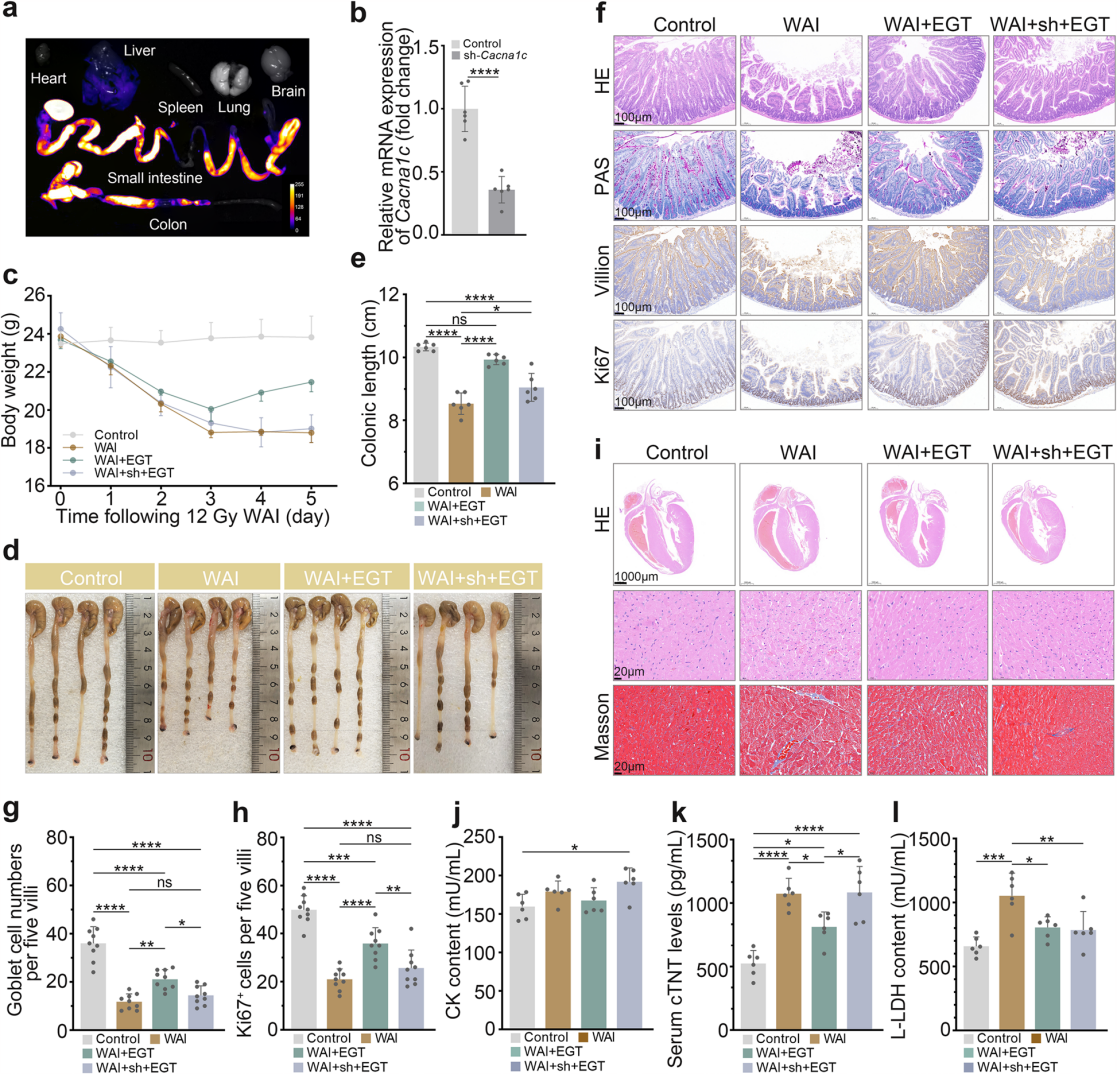
EGT protects against IR-induced myocardial injury via Cacna1c activation. **a** In vivo biodistribution of plasmid DNA assessed by bioluminescence imaging. **b** Relative mRNA expression of *Cacna1c* in the control group and WAI + sh + EGT group (*n* = 6). **c** Body weight changes post-IR. Representative colon images (**d**) and length quantification (**e**) (*n* = 6). **f** Representative images of small intestine tissue H&E, PAS and IHC staining (scale bar, 100 µm). **g** Goblet cells count per five villi. **h** Quantification of Ki67^+^ cells per five villi. **i** Representative images of cardiac tissue H&E and Masson staining (scale bar,1000 µm and 20 µm). Serum levels of cardiac injury markers CK (**j**), cTNT (**k**), and L-LDH (**l**) (*n* = 6). Data are shown as the means ± SD; ^*^P < 0.05, ^**^P < 0.01, ^**^P < 0.001, ^****^P < 0.0001

Genetic intervention studies revealed the critical role of Cacna1c in EGT-mediated radioprotection. Using orbital sinus injection of CACNA1C-targeting plasmid [21]. Bioluminescence imaging revealed predominant enrichment of Cacna1c in both small intestine and colon, while qRT-PCR and western blot analysis confirmed successful partial knockdown of CACNA1C expression (Fig. 4a, b and Fig. S2). In CACNA1C-deficient mice, EGT's therapeutic efficacy was substantially attenuated but not completely abolished: while weight loss remained unaffected (Fig. 4c), partial recovery of colon length was observed (Fig. 4d, e). Histological analysis showed, with EGT-treated knockdown mice exhibiting improved but incomplete restoration of villus architecture and goblet cell numbers compared to IR-only controls (Fig. 4f-h). Similarly, cardiac protection was partially maintained, with reduced myocardial damage (Fig. 4i) and moderate decreases in serum cardiac enzymes (Fig. 4j-l) despite CANCA1C knockdown. These findings demonstrate that while Cacna1c is a major mediator of EGT's effects, additional compensatory pathways are likely to contribute to its radioprotective activity.

### EGT mitigates WAI-induced cognitive deficits

**Fig. 5:**
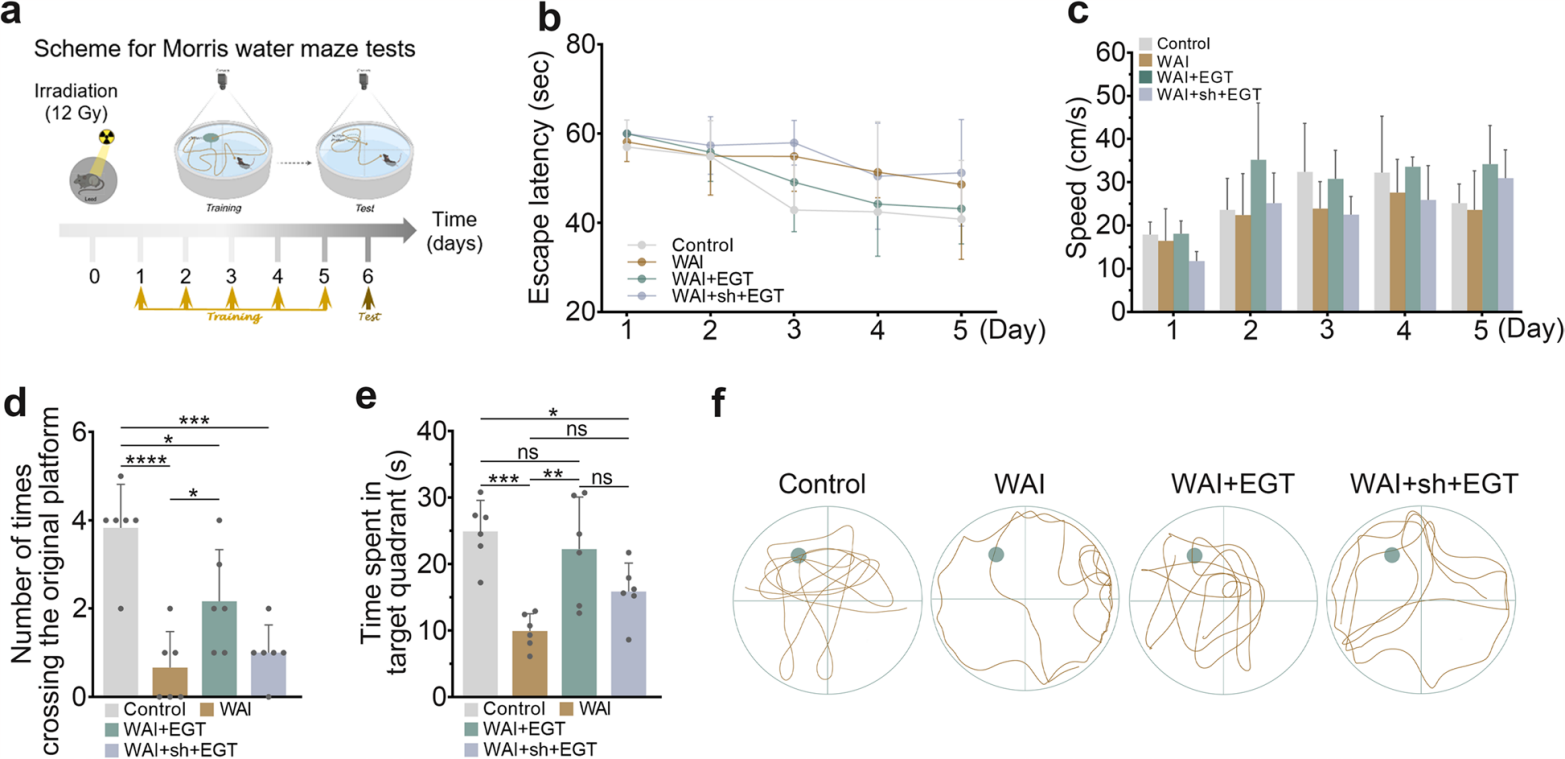
EGT mitigates WAI-induced cognitive deficits via gut-brain axis modulation. **a** Experimental timeline of WAI and Morris water maze tests protocol. **b** Escape latency during 5-day training (*n* = 6). **c** Swimming speed analysis (*n* = 6). Number of crossings through the former platform location (**d**) and time spent in the target quadrant (**e**) during the probe test on day 6 (*n* = 6). **f** Representative images of swimming trajectories. Data are shown as the means ± SD; ^*^P < 0.05, ^**^P < 0.01, ^**^P < 0.001, ^****^P < 0.0001

As previously demonstrated, WAI not only triggers myocardial injury through the gut-heart axis but also leads to neurological dysfunction and cognitive deficits in mice. To evaluate the therapeutic potential of EGT against WAI-induced cognitive impairment, we performed the Morris water maze test (Fig. 5a). The results revealed that WAI significantly extended the escape latency (time to reach the platform) and reduced swimming speed in mice. However, EGT treatment markedly shortened the escape latency and improved swimming speed, with effects surpassing those observed in CACNA1C knockdown mice (Fig. 5b, c). On the sixth testing day, compared to the control group, mice in the WAI and WAI + sh + EGT groups spent significantly less time in the target quadrant and exhibited fewer platform crossings. Notably, EGT administration reversed these deficits (Fig. 5d, e). Furthermore, swimming trajectory analyses further supported these findings (Fig. 5f). In conclusion, EGT mitigates WAI-induced cognitive dysfunction, likely through modulation of Cacna1c.

### EGT mitigates WAI-induced intestinal injury in female mice

**Fig. 6:**
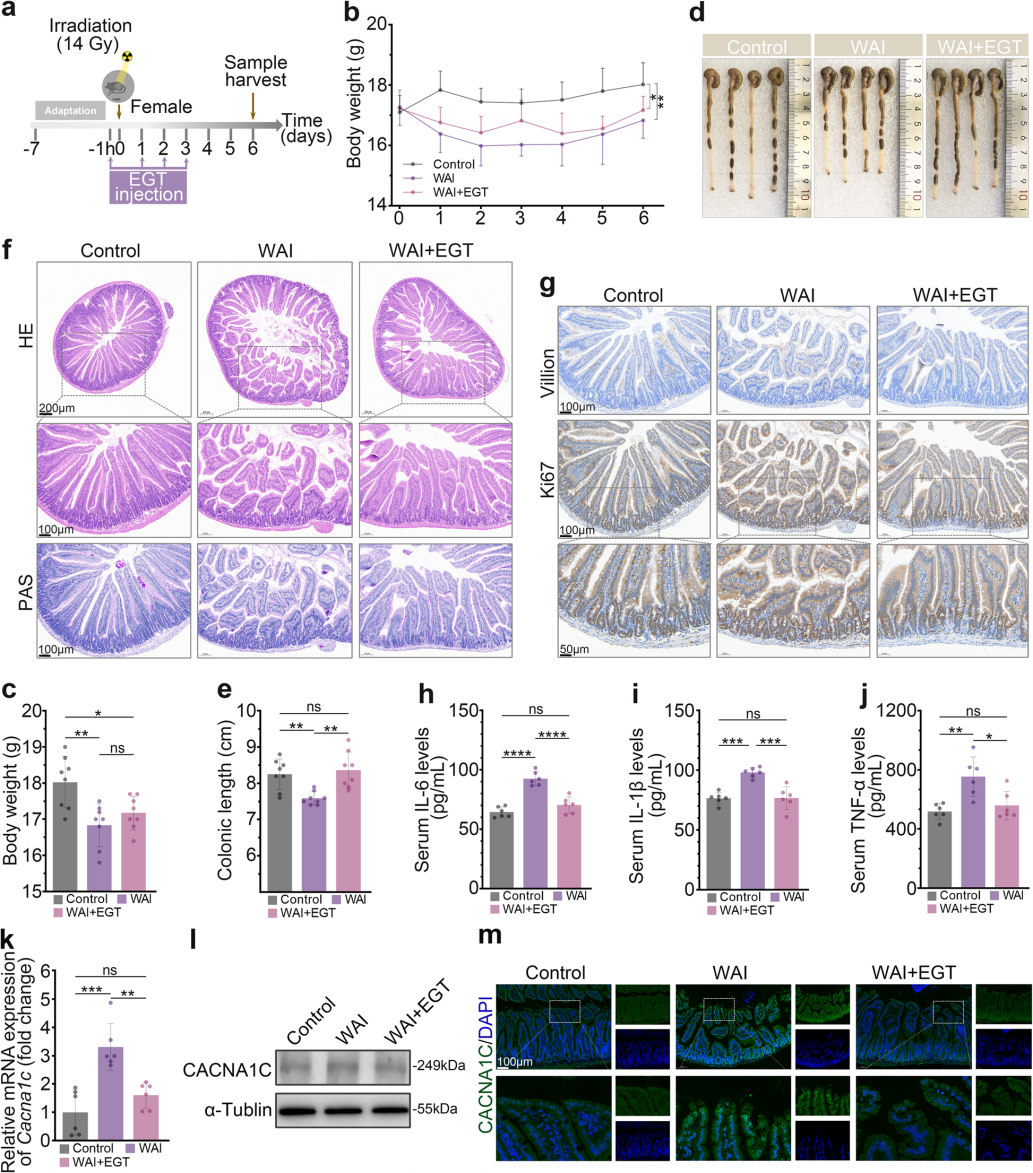
EGT attenuates WAI-induced intestinal injury in female mice. **a** Study design illustrating the 14 Gy WAI regimen and EGT administration schedule. **b**-**c** Body weight changes in female mice (*n* = 8). Representative colon images (**d**) and length quantification (**e**) (*n* = 8). **f** Representative images of small intestine tissue H&E and PAS (scale bar, 100 µm and 50 µm). **g** IHC detection of Ki67 and Villin in intestinal tissue (scale bar, 200 µm and 100 µm). Serum concentrations of pro-inflammatory cytokines IL-6 (**h**), IL-1β (**i**), and TNF-α (**j**) (*n* = 6). (**k**) qRT-PCR validation of mRNA expression (*n* = 6). **l** Protein level of CACNA1C. **m** Representative IHC images of Cacna1c expression (scale bar, 100 µm). Data are shown as the means ± SD; ^*^P < 0.05, ^**^P < 0.01, ^**^P < 0.001, ^****^P < 0.0001

Previous research has established significant sex-dependent differences in radiation response [22]. While our initial investigations employed male mice, we sought to determine whether EGT exerts comparable protective effects in females. To establish a gender-specific model of intestinal radiation injury with equivalent severity, and in light of our prior findings that female mice are less sensitive to IR than males, a higher radiation dose was administered to female mice [23]. Female mice received 14 Gy WAI followed by EGT treatment (40 mg/kg) administered 1 h prior to IR and continued for 3 days post-exposure (Fig. 6a). WAI exposure led to significant body weight loss in female mice, though EGT treatment failed to produce substantial weight recovery (Fig. 6b, c). However, EGT effectively prevented WAI-induced colon shortening (Fig. 6d, e). Histopathological examination revealed that WAI induced severe damage to small intestinal villi and crypts and significantly reduced goblet cell numbers, pathological changes that were substantially ameliorated by EGT treatment (Fig. 6f). IHC analysis showed that EGT significantly increased the number of Ki67^+^ and Villin^+^ cells compared to the WAI group (Fig. 6g). Like its effects in male mice, EGT treatment effectively normalized WAI-induced elevations in serum levels of IL-6, IL-1β, and TNF-α (Fig. 6h-j). Besides, our analysis revealed that EGT similarly suppresses IR-induced Cacna1c upregulation (Fig. 6k-m), mirroring the pattern observed in males. These findings collectively demonstrate that EGT's protective effects against WAI-induced intestinal damage are conserved across sexes, with no apparent gender-specific differences in therapeutic efficacy.

### EGT alleviates DSS-induced colitis in mice

**Fig. 7:**
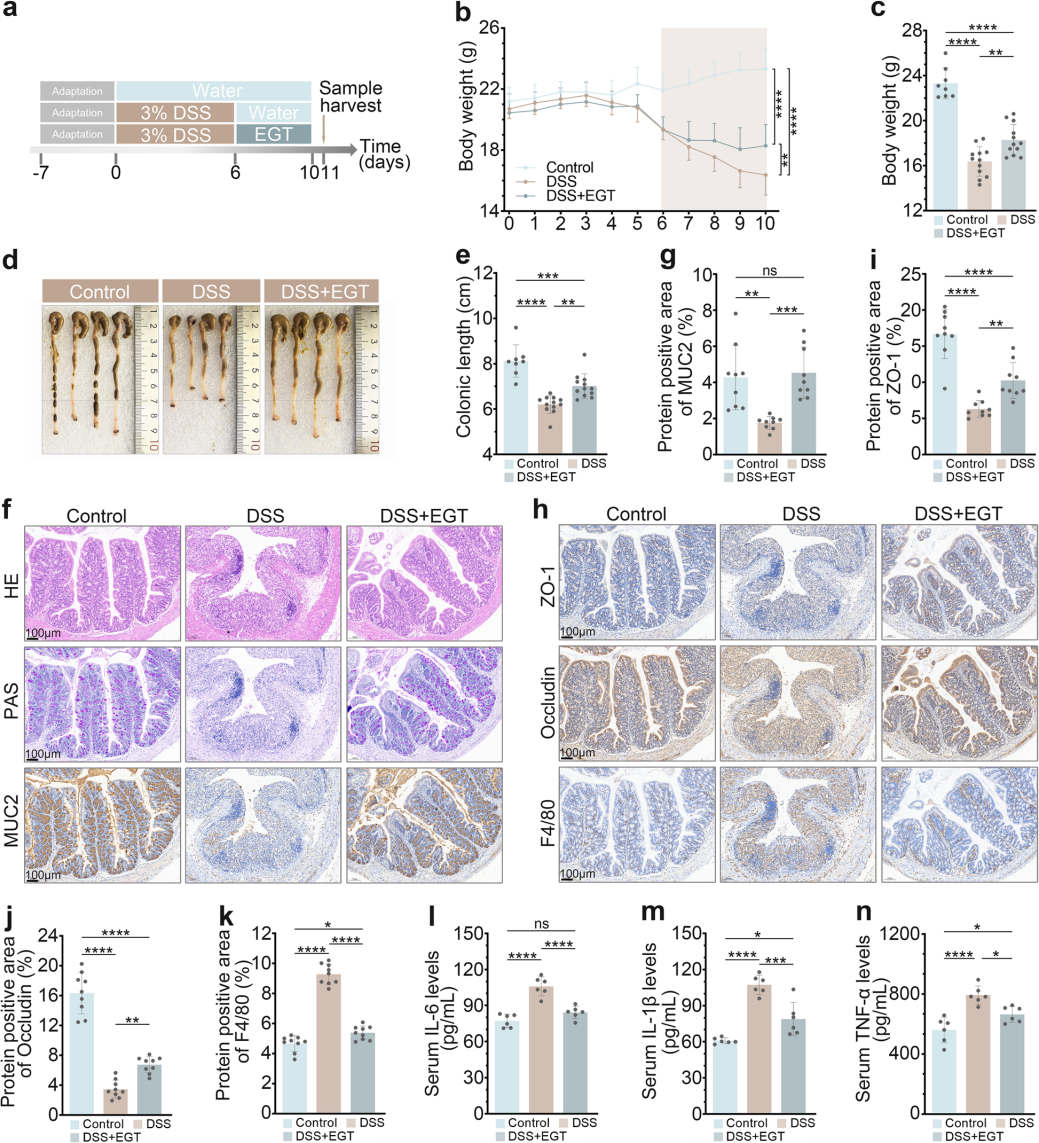
EGT alleviates DSS-induced colitis in mice. **a** Experimental design of DSS colitis induction and EGT treatment regimen. **b**-**c** EGT attenuated DSS-induced body weight loss (*n* = 8/12). Representative colon images (**d**) and length quantification (**e**) (*n* = 8/12). **f** Representative images of colon tissue H&E, PAS and MUC2 (scale bar, 100 µm). **g** Quantitative assessment of MUC2 expression. **h** Representative images of ZO-1, Occludin and F4/80 staining of colon tissue (scale bar, 100 µm). Quantitative analysis of ZO-1 (**i**), Occludin (**j**) and F4/80 (**k**) expression (*n* = 9). Serum concentrations of IL-6 (**l**), IL-1β (**m**), and TNF-α (**n**) (*n* = 6). Data are shown as the means ± SD; ^*^P < 0.05, ^**^P < 0.01, ^**^P < 0.001, ^****^P < 0.0001

To further evaluate the intestinal protective effects of EGT, we established a DSS-induced colitis model. Mice received 3% (w/v) DSS in drinking water during the experimental period (Fig. 7a), EGT treatment significantly attenuated DSS-induced progressive weight loss (Fig. 7b, c) and prevented DSS-mediated colon shortening (Fig. 7d, e). H&E and PAS staining examination demonstrated that EGT effectively mitigated DSS-induced intestinal damage, as evidenced by reduced inflammatory cell infiltration, preserved colonic gland architecture (Fig. 7f). Furthermore, IHC analysis indicated that EGT maintained the expression of MUC2, which is a major mucus barrier component produced by goblet cells, which was significantly suppressed in DSS-treated mice (Fig. 7f, g) [24]. Furthermore, EGT treatment reduced F4/80^+^ macrophage infiltration and restored the expression of tight junction proteins Zonula Occludens-1 (ZO-1) and Occludin (Fig. 7h-k). Serum analysis showed that EGT significantly lowered DSS-elevated levels of IL-6, IL-1β and TNF-α (Fig. 7l-n), confirming its systemic anti-inflammatory effects in this colitis model.

## Discussion

Pelvic and abdominal radiotherapy, a cornerstone for treating malignancies, are significantly limited by its toxicity. Up to 80% of patients develop acute radiation enteritis [25, 26], and abdominal irradiation can also induce remote cardiac injury and cognitive impairment via the enter cardiac axis [6, 9]. However, effective clinical radioprotectants are still lacking.

EGT, which is absorbed via the OCTN1 transporter, demonstrates biological benefits that extend beyond its relatively modest direct antioxidant capacity. It serves as an alternative substrate for cystathionine γ-lyase, thereby H_2_S production, enhancing protein persulfidation, and elevating NAD^+^ levels [10]. Previous research has shown that EGT promotes longevity in mice, and its endogenous levels inversely correlate with cognitive decline and cardiovascular pathologies [27–29], however, its potential role in IR-induced multi-organ damage remained unexplored until now.

Our current study establishes that EGT effectively alleviates WAI-induced intestinal injury in mice through coordinated anti-inflammatory and antioxidant mechanisms. Significantly, we found that EGT treatment downregulated intestinal Cacna1c expression and substantially reduced the abundance of the pathobiont *Candidatus_Soleaferrea*, a microorganism clinically associated with intestinal inflammation, cardiac diseases, and postoperative delirium [20, 30, 31]. While our study did not experimentally interrogate the causal role of *Candidatus_Soleaferrea* in IR-induced multi-organ injury, its established pro-inflammatory properties and correlation with systemic disorders support the plausibility that its suppression contributes to EGT's protective effects. We therefore propose that EGT mitigates IR-induced cardiac damage and cognitive impairment through modulation of the gut-heart-brain axis, mediated at least partially through reduction of *Candidatus_Soleaferrea*.

The Cacna1c encodes the core subunit of the L-type CaV1.2 channel, which is critical for cardiac excitation–contraction coupling and is implicated in intestinal motility and neuropsychiatric disorders [32–34]. By suppressing IR-induced Cacna1c upregulation, EGT modulates this key calcium signaling pathway to exert protection. EGT's efficacy was confirmed in both sexes, despite known sex-specific radiation responses [22].

Furthermore, EGT ameliorated DSS-induced colitis, reducing macrophage infiltration and restoring the expression of tight junction proteins ZO-1, Occludin and MUC2, confirming its broad anti-inflammatory and barrier-protective activities [35, 36]. Notably, EGT exhibits distinct effects on body weight in WAI and DSS models, highlighting the fundamentally different pathogenic mechanisms underlying these two forms of injury. In the WAI model, despite EGT's significant protective effects on intestinal integrity and inflammation, the severity and systemic nature of the injury limit its efficacy in fully restoring body weight. Conversely, in DSS-induced colitis, the more localized nature of the lesion allows EGT to directly maintain mucosal barrier function and nutrient absorption, thereby more effectively mitigating weight loss. This contrast underscores the context-dependent therapeutic potential of EGT and indicates that its efficacy in alleviating weight loss is closely tied to the specific pathophysiology of intestinal injury.

Study limitations include the partial reversal of protection by CACNA1C knockdown, suggesting additional mechanisms, and the need to establish causality between microbial shifts and organ protection using germ-free models. Future work will focus on EGT's direct effects on CaV1.2 channel function and identifying other key mediators within the gut-heart-brain axis under clinically relevant, fractionated radiation.

## Conclusion

In summary, this study demonstrates that EGT ameliorates WAI-induced multi-organ injury through gut microbiota and Cacna1c-mediated mechanisms. Using a murine WAI model combined with multi-omics approaches, we found that EGT not only alleviated intestinal structural damage and promoted barrier recovery but also remodeled gut microbiota composition, specifically reducing the abundance of *Candidatus_Soleaferrea*. It also downregulated Cacna1c expression. Furthermore, EGT exerted cardioprotective and cognitive-preserving effects, likely mediated through the gut-heart and gut-brain axes. These findings, together with its efficacy in mitigating DSS-induced colitis, establish EGT as a promising broad-spectrum radioprotective agent with therapeutic potential for clinical radiotherapy-related injury.

## Materials and methods

### Animals

Six-to-eight-week-old male C57BL/6J mice were obtained from Beijing Huafukang Biotechnology Co., Ltd. (Beijing, China) and maintained in specific pathogen-free (SPF) conditions at the Experimental Animal Center of the Institute of Radiation Medicine, Chinese Academy of Medical Sciences (IRM-CAMS). Animals were housed under controlled environmental conditions (temperature: 22 ± 1 °C; humidity: 50 ± 5%; 12 h/12 h light/dark cycle) with ad libitum access to standard chow and autoclaved water. Following a 7-day acclimatization period, experimental procedures were initiated. All animal experiments conducted in this study were carried out in accordance with a protocol approved by the Institutional Animal Care and Use Committee of IRM, CAMS.

### Irradiation and EGT administration

WAI was performed using a ^137^Cs γ-ray irradiator (Gammacell-40; Atomic Energy of Canada Ltd., Chalk River, ON, Canada) with a calibrated dose rate of 0.8 Gy/min. Male mice received 12 Gy and female mice received 14 Gy WAI. Prior to irradiation, animals were anesthetized with isoflurane and positioned in prone orientation, with custom lead shielding applied to protect non-targeted body regions. EGT (HY-N1914, MedChemExpress) was dissolved in sterile distilled water to prepare working concentrations of 20, 40, and 80 mg/kg. For dose optimization studies, mice received an initial intraperitoneal injection of EGT 1 h prior to irradiation followed by three consecutive daily injections post-irradiation. Control mice were sham-irradiated. Control group and irradiation group mice were given the same volume of sterile distilled water via intraperitoneal injection. The complete experimental timeline is illustrated in Fig. 1a.

### DSS colitis model

Acute colitis was induced by administering 3% (w/v) DSS (Yeasen Biotech, China) in drinking water ad libitum for 5 days. Treatment groups received daily intraperitoneal injections of EGT at the predetermined optimal concentration throughout the DSS exposure period. Intestinal tissues were collected on day 5 and preserved in 4% paraformaldehyde for further examination.

### Plasmid construction, injection, and bioluminescence imaging

Knockdown of CACNA1C was performed using a specific shRNA, which was cloned into the pRNA-U6.1/Neo plasmid by Vector Builder (Guangzhou, China). The target sequences of sh-CACNA1C used in this study are listed in Supplementary Table 1. Plasmid delivery and subsequent biofluorescence imaging were performed following previously described protocols [23, 37]. Mice were anesthetized with isoflurane, placed on their sides with the eyes protruding, and the plasmid was rapidly injected into the retroorbital sinus. Mice were euthanized 24 h later, organs were removed, and images were acquired using the IVIS Lumina imaging system (IVIS Lumina II, PerkinElmer). Fluorescence signal intensity was measured using ImageJ software.

### Bacterial diversity analysis

To assess the effects of WAI and EGT treatment on the gut microbiota, we collected fresh fecal samples from mice for bacterial diversity analysis. As previously described, DNA library preparation and analysis of the 16S rRNA V4 gene were performed (Novogene Bioinformatics Technology, China). The data from each sample were then normalized, and subsequent alpha diversity analysis and beta diversity analysis were based on the normalized data [38].

### RNA-seq

Total RNA was extracted from small intestine tissue. After RNA integrity was verified, library construction and sequencing were performed using the Illumina sequencing platform (Novogene Bioinformatics Technology, China). Principal component analysis, correlation analysis, GO enrichment analysis, and KEGG pathway enrichment analysis were performed based on gene expression levels.

### Morris water maze tests

Spatial learning and memory were assessed using a standard water maze protocol. Mice were trained for 5 consecutive days (3 trials/day) to locate a hidden platform in a circular pool (120 cm diameter) maintained at 22 ± 2℃. Escape latency and swim speed were recorded using EthoVision XT (Noldus, The Netherlands). On day 6, memory retention was evaluated in a 60s test without the platform, measuring time in target quadrant and platform crossings. The experimental timeline is shown in Fig. 5a.

### Statistical analysis

All data in this study were analyzed using Prism 9 software. Student's t-test or one-way analysis of variance (ANOVA) were used for analysis, and data are expressed as mean ± standard deviation (SD). Results with *P* < 0.05 were considered statistically significant.

## Supplementary Information

Supplementary Material 1.

## Data Availability

The RNA- sequencing data have been deposited in the National Genomics Data Center, China National Center for Bioinformation (CNCBNGDC) database (https://ngdc.cncb.ac.cn/, accession number: CRA034655). The datasets generated and/or analyzed during this study are available within the article and its supplementary information files. Additionally, other relevant data may be obtained from the corresponding author upon reasonable request.

## Abbreviations

EGT: Ergothioneine
IR: Ionizing radiation
IHC: Immunohistochemistry
RIHD: Radiation-induced heart disease
ROS: Reactive oxygen species
PAS: Periodic acid-Schiff
WAI: Whole abdominal irradiation
H&E: Hematoxylin and eosin
PCoA: Principal coordinate analysis
CaV1.2: L-type voltage-gated calcium channel 1.2
cTNT: Cardiac troponin T
CK: Creatine kinase
L-LDH: L-Lactate dehydrogenase
