# Supplementary material for: Ergothioneine attenuates whole-abdominal irradiation-induced multi-organ injury via the gut-heart-brain axis by modulating calcium voltage-gated channel subunit alpha1 C (Cacna1c) expression
Source: Mol Biomed. 2026 Jan 14;7:6. doi: 10.1186/s43556-025-00402-3 (PMC12799843; doi:10.1186/s43556-025-00402-3)
Supplement: Supplementary file 1 — Supplementary Material 1. [file 43556_2025_402_MOESM1_ESM.docx]

**Supplementary Information**

**Ergothioneine attenuates whole-abdominal irradiation-induced multi-organ injury via the gut-heart-brain axis by modulating** **calcium voltage‑gated channel subunit alpha1 C (Cacna1c) expression**

Xudong Ding^1^, Jia Du^1^, Zhaoyu Wang^1^, Lu Lu^1, *^, Saijun Fan^1, *^

^1^ Institute of Radiation Medicine, Chinese Academy of Medical Sciences and Peking Union Medical College, Tianjin Key Laboratory of Radiation Medicine and Molecular Nuclear Medicine, State Key Laboratory of Advanced Medical Materials and Devices, Tianjin 300192, China

***Corresponding authors:**

Lu Lu, lulu@irm-cams.ac.cn

Saijun Fan, fansaijun@irm-cams.ac.cn

**Supplementary Methods**

**Histopathology**

Tissue samples (small intestine, colon, and heart) were fixed in 4% paraformaldehyde, paraffin-embedded, and sectioned at 3-5 μm thickness. Sections were stained with hematoxylin and eosin (H&E) for general morphological evaluation. Heart tissues were additionally stained with Masson's trichrome to assess fibrosis, while intestinal tissues underwent periodic acid-Schiff (PAS) staining to evaluate mucosal integrity. All stained sections were digitally scanned using a Pannoramic P250 scanner (3DHISTECH, Hungary) and analyzed using CaseViewer software.

**Immunohistochemistry (IHC)**

Intestinal and colon tissue sections were dewaxed and rehydrated, then incubated with anti-Cacna1c antibody (OM629068; Omnimabs), anti-Villin antibody (ab130751; Abcam), anti-Ki67 antibody (ab15580; Abcam), anti-MUC2 antibody (GB11344; Servicebio), anti-ZO-1 antibody (GB111402; Servicebio, China), anti-Occludin antibody (GB111401; Servicebio, China), and anti-F4/80 antibody (GB113373; Servicebio, China). The sections were then incubated with biotin-labeled secondary antibodies and observed with a Pannoramic P250 scanner (3DHISTECH, Hungary) and analyzed using CaseViewer software. Three sections of small intestinal or colonic tissue were stained per group. Positive staining was quantified by performing three counts per section.

**Quantitative real-time PCR (qRT-PCR)**

RNA was extracted from small intestine tissue using Trizol and reverse transcribed using the PrimeScript™ RT Kit (RR037A; Takara) according to the manufacturer's protocol. Real-time PCR was performed using SYBR Green qPCR Premix (Universal) (MCE). The fold change in target gene expression was calculated using the 2^−ΔΔCq^ method based on the Cq values detected by the QuantStudio™ 6 Flex System (Thermo). Primer sequences are shown in Supplementary Table 2.

**Western blot analysis**

Small intestinal tissue was homogenized using RIPA lysis buffer, protease inhibitor, and phosphatase inhibitor in specified proportions. After homogenization, the tissue was centrifuged for 15 minutes to collect the supernatant. The supernatant was mixed with SDS-PAGE protein loading buffer at a 4:1 ratio and denatured at 37°C for 30 minutes. Proteins were separated using 6% or 10% SDS-PAGE gels and transferred to polyvinylidene fluoride (PVDF) membranes. After blocking with TBS blocking bufffer (37579; Thermo Scientific) at room temperature for 15 minutes, membranes were incubated with primary antibodies Alpha-Tubulin (BOAM-D0276-A; Biosharp), Cacna1c (ACC-003; Alomone Labs), followed by incubation with the corresponding secondary antibody. All blots were developed using ECL chemiluminescent reagents and analyzed with Image Lab software.

**Enzyme-linked immunosorbent assay (ELISA)**

Peripheral blood was collected from the orbital sinuses of mice and centrifuged at 4000 rpm at 4 °C for 10 minutes to collect the supernatant serum. Following the product instructions, use the ELISA kit (Tongwei, Shanghai, China) to detect the levels of IL-1β, IL-6, and TNF-α in the serum. Additionally, use the Cardiac troponin T (cTNT) detection kit (SEKM-1050, Solarbio), Creatine kinase (CK) detection kit (S0287S, Beyotime Biotechnology), and L-Lactate dehydrogenase (L-LDH) detection kit (P0393S, Beyotime Biotechnology) to measure the levels of cardiac enzymes in serum.

**Supplementary Table 1.sh-Cacna1c sequence**

| **Gene** | **Sequence (5′−3′)** |
| --- | --- |
| Sh-CACNA1C-1 | TACAACCAGGAGGGCATAATACTCGAGTATTATGCCCTCCTGGTTGTA |
| Sh-CACNA1C-2 | TTCAAACCCAAGCACTATTTCCTCGAGGAAATAGTGCTTGGGTTTGAA |

**Supplementary Table 2. Primer sequences**

| **Gene** | **Primer** | **Sequence (5′−3′)** | **Product length (bp)** |
| --- | --- | --- | --- |
| *Cacna1c* | Forward | ATGAGACCCGCAGCGTAAGG | 175 |
|  | Reverse | GAGGCAGAGCGAAGGAAACT |  |
| *GAPDH* | Forward | TGGAAGGGCTCATGACCACAG | 224 |
|  | Reverse | CAGATCCACGACGGACACATT |  |

**Supplementary figures and figure legends**

**Fig.S1**

**
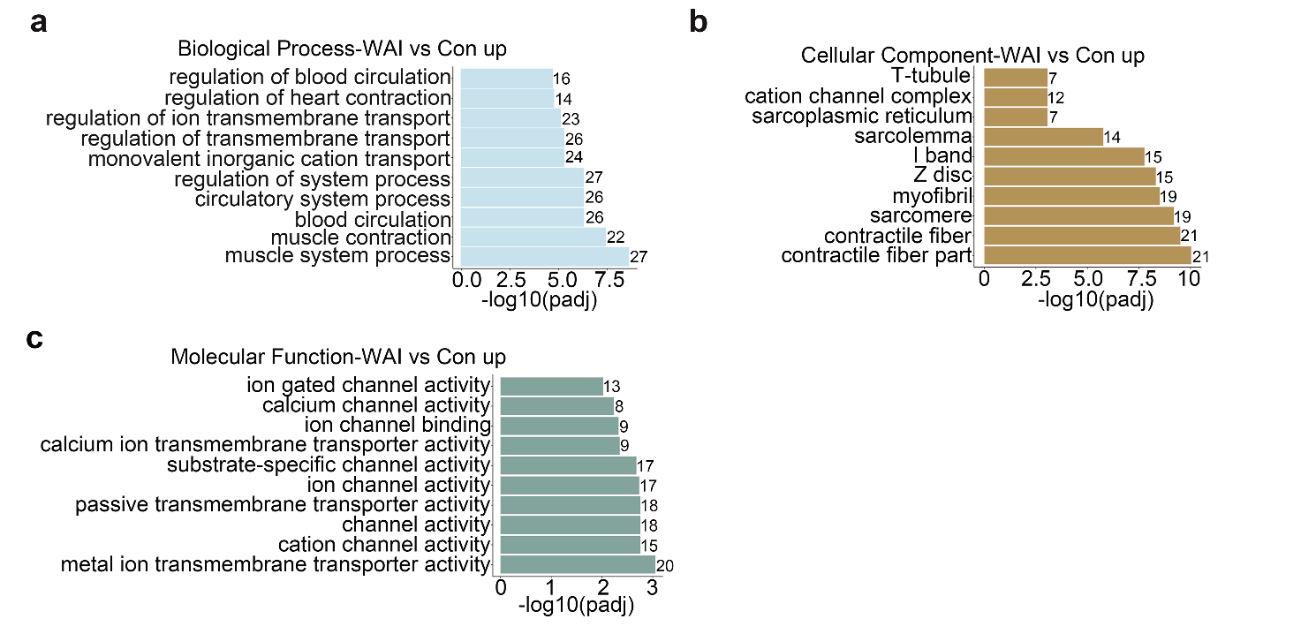
**

**Fig. S1** **GO enrichment analysis between the control group and the WAI group**. Enrichment results of differentially upregulated genes between the control group and the WAI group in Biological Process (a), Cellular Component (b), and Molecular Function (c).

**Fig.S2**


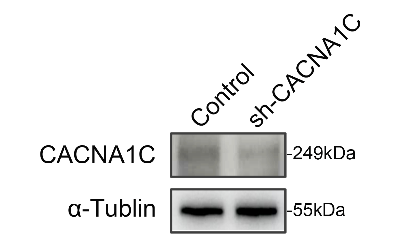


**Fig. S2 Protein level of** **CACNA1C between the control group and the sh-CACNA1C group.**
